# A Hybrid Gene Selection Method Based on ReliefF and Ant Colony Optimization Algorithm for Tumor Classification

**DOI:** 10.1038/s41598-019-45223-x

**Published:** 2019-06-20

**Authors:** Lin Sun, Xianglin Kong, Jiucheng Xu, Zhan’ao Xue, Ruibing Zhai, Shiguang Zhang

**Affiliations:** 1 College of Computer and Information Engineering, Henan Normal University, Xinxiang, 453007 China; 2 Post-doctoral Mobile Station of Biology, College of Life Science, Henan Normal University, Xinxiang, China; 3 School of Computer Science and Technology, Tianjin University, Tianjin, 300072 China

**Keywords:** Classification and taxonomy, Computational models, Data processing, Machine learning

## Abstract

For the DNA microarray datasets, tumor classification based on gene expression profiles has drawn great attention, and gene selection plays a significant role in improving the classification performance of microarray data. In this study, an effective hybrid gene selection method based on ReliefF and Ant colony optimization (ACO) algorithm for tumor classification is proposed. First, for the ReliefF algorithm, the average distance among *k* nearest or *k* non-nearest neighbor samples are introduced to estimate the difference among samples, based on which the distances between the samples in the same class or the different classes are defined, and then it can more effectively evaluate the weight values of genes for samples. To obtain the stable results in emergencies, a distance coefficient is developed to construct a new formula of updating weight coefficient of genes to further reduce the instability during calculations. When decreasing the distance between the same samples and increasing the distance between the different samples, the weight division is more obvious. Thus, the ReliefF algorithm can be improved to reduce the initial dimensionality of gene expression datasets and obtain a candidate gene subset. Second, a new pruning rule is designed to reduce dimensionality and obtain a new candidate subset with the smaller number of genes. The probability formula of the next point in the path selected by the ants is presented to highlight the closeness of the correlation relationship between the reaction variables. To increase the pheromone concentration of important genes, a new phenotype updating formula of the ACO algorithm is adopted to prevent the pheromone left by the ants that are overwhelmed with time, and then the weight coefficients of the genes are applied here to eliminate the interference of difference data as much as possible. It follows that the improved ACO algorithm has the ability of the strong positive feedback, which quickly converges to an optimal solution through the accumulation and the updating of pheromone. Finally, by combining the improved ReliefF algorithm and the improved ACO method, a hybrid filter-wrapper-based gene selection algorithm called as RFACO-GS is proposed. The experimental results under several public gene expression datasets demonstrate that the proposed method is very effective, which can significantly reduce the dimensionality of gene expression datasets, and select the most relevant genes with high classification accuracy.

## Introduction

Over the few decades, bioinformatics has become a more and more notable research field since it allows biologists to make full use of the technologies in computer science and computational statistics to analyze the data of an organism at the genomic, transcriptomics and proteomic levels^1–3^. One of the major tasks in biomedicine is the classification and the prediction of microarray data^3^. With the rapid development of DNA microarray technology, classification of microarray data is a challenging task since gene expression datasets are often with thousands of genes but a small number of samples^4^. Tumor classification is one of the conventional problems in microarray gene expression data, and includes tumor detection and prediction of some rare diseases^5^. These studies are of tremendous importance for accurate cancer diagnosis and subtype recognition. Because of the limited availability of effective samples compared to thousands or even tens of thousands of genes in microarray data, many computational methods fail to identify a small portion of important genes, and it increases learning costs and deteriorates learning performance^6,7^. In general, cancer classification for microarray data involves data collection, preprocessing, gene selection, and so on. The goal of classification is to build efficient and effective gene selection methods, which reduce the dimensionality of microarray data to improve the classification accuracy of cancer gene expression datasets. The aim of gene selection is to reduce the dimensionality of microarray data in order to enhance the accuracy of classification task^8^. Gene selection methods can reduce the number of irrelevant and noisy genes and select the most related genes to improve the classification results, which decrease the computational costs and improve the cancer classification performance^9^.

The methods applied for feature selection are broadly divided into four categories including the filter, the wrapper, the embedded and the hybrid approaches^10–15^. Considering independence of the classifier, the filter methods have been widely used because of the advantage of high speed and effectively dealing with large datasets^16^, but they are easily trapped into local optimum. Sun *et al*.^17^ raised a cross-entropy-based multi-filter ensemble method for gene selection. Though the wrapper methods contain a given learning model, they suffer from the high computational cost, particularly on the high-dimensional microarray datasets. Wang *et al*.^18^ presented a wrapper-based gene selection method by introducing the Markov blanket technique to reduce the required wrapper evaluation time. This wrapper approach achieved superior classification accuracy, but need much more computational power^19^. For the embedded methods, training a given classifier with a full feature set is time-consuming. Lopes *et al*.^20^ proposed an ensemble classification setting based on distinct feature selection and modeling strategies to classify the breast cancer data. Li *et al*.^21^ developed an embedded feature selection algorithm that could adaptively identify important features through introducing data driven weights. However, this method requires adjusting more parameters, and its performance largely depends on those parameters. The major disadvantage of hybrid approaches is that the filter and wrapper approaches do not integrate with each other well, which may result in the lower classification accuracy^16^. Mav *et al*.^22^ presented a hybrid gene selection approach to create the targeted gene sets in high-throughput transcriptomics. Overall, the framework can be designed to combine their virtues of both the filter and the wrapper methods for feature selection to get an efficient and accurate approach^23^. Algamal *et al*.^24^ developed a gene expression classification approach using Bayesian Lasso quantile regression for the sake of overcoming the sensibility of outliers in gene data. Lin^25^ proposed a gene selection scheme to generate multiple subsets with variable gene combinations supporting classification tasks. Our gene selection method is based on the hybrid approach, in which a search algorithm is used to find an optimal gene subset for gene expression datasets.

Recently, the Relief algorithm, as a high-efficiency filter approach, is performed well^26^. However, the classical Relief method cannot handle the issue of missing data and noise. Kononenko^27^ extended Relief as the ReliefF algorithm, which can solve multi-classification, data missing and the existence of noise and other issues^28^. Wang and Gong^29^ combined ReliefF and minimal-Redundancy-Maximal-Relevance (mRMR) to reduce feature dimensionality, but its process of implementation is complex. Liu *et al*.^30^ developed a gene selection method based on ReliefF and particle swarm optimization. Wang *et al*.^31^ studied a mean deviation-based sample weighting versions of ReliefF, which can improve the stability of the results of feature selection. Lu *et al*.^32^ constructed a gene selection method combining ReliefF and the extreme leaning machine (ELM) as the classifier. Through analyzing the abovementioned Relief methods, the selection of nearest neighbor samples is the basic difference between Relief and ReliefF. Furthermore, the robustness of ReliefF can be effectively indicated by averaging the difference of nearest neighbor samples. The series Relief algorithms based on feature weights have low time complexity. However, the redundant features using the ReliefF algorithm cannot be removed by excluding features with low weights. What’s more, there exist still some disadvantages for the ReliefF algorithm. For example, the randomly selected samples are less when finding out the nearest neighbor samples and the feature weights have large fluctuations. To address these issues, this paper focuses on improving the ReliefF algorithm.

Compared with the filter methods, the wrapper models select feature subsets with higher prediction accuracy with some search methods, and then their results are evaluated by a certain learning algorithm^33^. The search methods in wrapper are divided into sequential strategies and random strategies. Recently, some wrapper-based approaches have been provided and widely applied in bioinformatics, such as genetic algorithm^34^ (GA), particle swarm optimization^35^ (PSO), Ant colony optimization^36^ (ACO), and so on. Although these approaches have obtained excellent performance in gene expression data analysis, some congenital drawbacks still puzzle themselves such as excessive computational cost of GA and local optimum of PSO^36^. The ACO algorithm is inspired by the behavior of real ants. Since ACO needs no heuristic information for searching an optimal minimal subset every time, it is especially an attractive approach to feature selection^37^. The ACO-based feature selection enables to efficiently balance between exploration and exploitation, and then can find more important features by taking advantage of the parameter adjustment and feature significance^38^. It has intelligent searching, global optimization, robustness and positive feedback; so many scholars have paid more attention to ACO^39^. Thus, ACO is more suitable to handle high-dimensional, noise, irrelevant and redundant dataset than PSO and GA^40^. Until now, the ACO algorithm has been successfully applied to solve different applications such as data mining, classification, bioinformatics and etc.^36–42^. Shi *et al*.^41^ designed an ensemble algorithm for biomedical classification based on ACO. Yu *et al*.^36^ improved the ACO algorithm to select tumor-related marker genes. Cui *et al*.^42^ presented an ACO-based method for gene selection to get better classification accuracy. However, some of these methods have the following shortcomings: (1) The convergence speed is too slow. (2) It is easy to fall into the local optimum. (3) The pheromone is overwhelmed with time. These issues inspire the authors to investigate and improve the nature inspired optimization algorithm about ACO in this paper.

During the previous years, by integrating the complementary strengths of filter and wrapper approaches well, some hybrid methods have been developed to select the significant features and balance the relationship between efficiency and accuracy for selecting an optimal feature subset^43^. For example, Xiong and Wang^44^ proposed an ACO and random forest-based hybrid search method, which improved the ability to traverse the search space and select feature subsets. The integrated method can efficiently improve the efficiency and the accuracy of feature selection to some extent^45^. Then, the objective of this paper is to combine the ReliefF algorithm with the ACO algorithm to develop a hybrid filter-wrapper search technique for gene selection, where the ReliefF algorithm, as a filtering approach, eliminates some less relevant genes, and the ACO algorithm search the top-rated genes and further select the most useful genes that can perform accurate cancer classification. Firstly, the improved ReliefF is used to calculate the weights of each gene that are sorted in descending order. Then, the candidate genes are selected according to the weights, and the new pruning rule for the ACO algorithm is used to retain the genes whose weights are larger than the average value, which can accelerate the calculation. The improved probability formula of candidate genes is proposed, which can highlight the closeness between variables and increase the path visibility. The pheromone updating rule is used to increase the pheromone concentration of important genes, which can make the search results more reasonable and not deviate from the actual situation. Finally, the integration of the improved ReliefF algorithm and the improved ACO algorithm results in an effective gene selection method. The experiments show that this method can effectively remove the irrelevant and redundant genes of classification data and improve the classification performance.

The remainder of this paper is structured as follows. In Section 2, some related studies of ReliefF and ACO are recalled. The improved ReliefF method, the improved ACO method and the RFACO-GS algorithm are described in Section 3. The experimental results and analysis of gene expression datasets are shown in Section 4. In Section 5, the conclusions are given.

## Related Studies

### The ReliefF Algorithm

ReliefF algorithm is one of the widely applied filter-based feature selection models and has great classification efficiency. In addition, this algorithm does not limit data types and can effectively deal with nominal or continuous features, missing data and noisy tolerance^46^. The principle of this algorithm is that the stronger correlation of classification makes the similar samples closer. On the contrary, the inhomogeneous samples are kept away.

The detailed operation steps of the ReliefF algorithm^47^ can be described as follows: Firstly, a sample *x*_*i*_ is selected from the training samples, *k* nearest neighbor samples of *x*_*i*_ are selected and written as *H*, and then *k* non-similar nearest neighbor samples of the different class from *x*_*i*_ are selected and written as *M*(*c*). In order to adjust the weight vectors of features, the feature weights are obtained by calculating the within-class and the between-class distances of the nearest neighbor samples. The weights of all features are eventually yielded by repeating this procedure.

The formula of updating the weight value of features by the ReliefF algorithm is expressed as

1 $$W[A]=W[{A}_{0}]-\frac{\sum _{j=1}^{k}diff(A,{x}_{i},H)}{mk}+\sum _{C\ne class({x}_{i})}\frac{p(C)}{1-p(class({x}_{i}))}\cdot \frac{\sum _{j=1}^{k}diff(A,{x}_{i},{M}_{j}(C))}{mk},$$

where *A*_0_ is a feature set of the original dataset; *A* represents a feature subset of the filtered dataset; *W*[*A*_0_] acts for the weight coefficient before updating; *W*[*A*] stands for the updated weight coefficient; *x*_*i*_ is the *i-*th sample and *H* represents the nearest neighbor samples with *x*_*i*_ in the same class; *diff*(*A*, *x*_*i*_, *H*) is a quantitative representation of the difference between *x*_*i*_ and *H* on each feature in *A*; *m* is the number of the cumulative repeats; *k* is the number of the nearest neighbors; *p*(*C*) is the ratio of the target samples *C* to the total samples; *p*(*class*(*x*_*i*_)) is the ratio of the samples in the same class including *x*_*i*_ to the total samples; *M*_*j*_(*C*) denotes the *j*-th neighbor sample in the different class with the target samples *C*; and *diff*(*A*, *x*_*i*_, *M*_*j*_(*C*)) is a quantitative representation of the difference between *x*_*i*_ and *M*_*j*_(*C*) on each feature in *A*.

### The ACO algorithm

ACO algorithm is one of the applications of wrapper-based feature selection methods and a probabilistic technique for solving computational problems to reduce the search path to find the optimal path through graphs, which can be usually used to find an optimum subset of features^48^. The ACO algorithm has the strong robustness and the great performance on resolving the complex optimization problem, and is state-of-the-art for addressing the optimization problem of feature selection. It requires a problem that can describe a graph, where the nodes indicate features with edges among nodes and describe the next option of feature^49^. This optimal feature subset search is an ant path through graph where the minimum number of the visited nodes is suitable with the traversal stopping criterion^48^.

Let *τ*_*ij*_(0) = *C*, where *C* is a constant, and the *k*-th ant decides the direction according to the number of pheromones on each path, where *k* = 1, 2, …, *m*. The probability of the *k*-th ant shifts from the *i-*th position to *j-*th position at *t*-th moment, which is described as

2 $${p}_{ij}^{k}(t)=\frac{{\tau }_{ij}^{\alpha }(t){\eta }_{ij}^{\beta }(t)}{{\sum }_{s\in allowe{d}_{k}}{\tau }_{ij}^{\alpha }(t){\eta }_{is}^{\beta }(t)},$$

where *α* stands for the relative importance of the track and *α* > 0; *β* acts for the relative importance of visibility and *β* ≥ 0; *ρ* is the retain ability of the track and stands for the attenuation degree, and 0 < *ρ* < 1; and *η*_*ij*_ is the visibility of arc (*i*, *j*) and can be calculated by us*i*ng a meta-heuristic algorithm^50^, which is usually expressed as

3 $${\eta }_{ij}=\frac{1}{{d}_{ij}},$$

where *d*_*ij*_ is the distance between the *i*-th node and the *j*-th node.

After a certain time, Δ*t* has elapsed and the ant finishes one cycle, then the information amounts for each path are adjusted by

4 $${\tau }_{ij}(t+{\rm{\Delta }}t)=(1-\rho ){\tau }_{ij}(t)+{\rm{\Delta }}{\tau }_{ij}(t),$$

5 $${\rm{\Delta }}{\tau }_{ij}(t)={\sum }_{k=1}^{m}{\tau }_{ij}^{k}(t),$$

where ${\tau }_{ij}(t+{\rm{\Delta }}t)$ is an updated pheromone value of the *i*-th feature and the *j*-th feature; *τ*_*ij*_(*t*) is the number of the residual pheromones on (*i*, *j*) a*t t*-th moment; ${\tau }_{ij}^{k}(t)$ represents the information amount of path *i*, *j* left in this cycle; and Δ*τ*_*ij*_ is the information gain for path *i*, *j* for this cycle.

${\rm{\Delta }}{\tau }_{ij}^{k}(t)$ is the sum of the pheromones remaining in the cycle of the *k*-th ant^40^, which can be calculated by

6 $${\rm{\Delta }}{\tau }_{ij}^{k}(t)=\frac{Q}{{L}_{k}},$$

where *Q* is the amount of pheromone on the path from Ants in the iteration, and *L*_*k*_ is the fitness function that is the path length for one travel cycle, which is described as

7 $${L}_{{\rm{k}}}=\sum _{j=1}^{n}D(R(j),\,R(j+1)),$$

where *R*(*j*) represents the location of the *j*-th feature, and *D*(*R*(*j*), *R*(*j* + 1)) is the path length between two feature point location with the Euclidean distance.

## Proposed Hybrid Gene Selection Method for Tumor Classification

### Improved relieff method

The ReliefF algorithm, as a kind of feature estimator, can efficiently offer quality measures of features in handling the complex problems with strong dependencies among features^46^. For the classification based on gene expression data, the goal of ReliefF algorithm for gene selection is to evaluate the quality of genes according to how well their values distinguish between samples that are near to each other. In order to effectively reduce the redundancy in selecting genes and further enhance the classification accuracy of the selected genes, the ReliefF algorithm is improved to measure the gene weight for tumor classification.

**Definition 1**. The distance between the sample *x*_*i*_ and the samples in the same class with *x*_*i*_ on the gene subset *A* is defined as

8 $$dis(A,{x}_{i},H)=\sum _{i=1}^{k}\frac{|{x}_{i}-\overline{H}|}{\max (A)-\,\min (A)},$$

where *H* represents the samples in the same class with *x*_*i*_; $\bar{H}$ is the average distance among *k* nearest neighbor samples in the same class with *x*_*i*_; max(*A*) describes the maximal feature value of gene subset *A*; and min(*A*) represents the minimal feature value of gene subset *A*.

**Definition 2**. The distance between the sample *x*_*i*_ and the samples *M*_*j*_(*C*) in the different classes with *x*_*i*_ on the gene subset *A* is defined as

9 $$dis(A,{x}_{i},{M}_{j}(c))=\sum _{c\ne class({x}_{i})}\frac{p(C)}{1-p(class({x}_{i}))}\sum _{i=1}^{k}\frac{|{x}_{i}-\overline{{M}_{j}(C)}|}{\max (A)-\,\min (A)},$$

where *p*(*C*) is the ratio of the target samples *C* to the total samples; *p*(*class*(*x*_*i*_)) is the ratio of the samples of classes including *x*_*i*_ to the total samples; $\overline{{M}_{j}(C)}$ is the average distance among *k* non-nearest neighbor samples in the different classes with *x*_*i*_; max(*A*) describes the maximal feature value of gene subset *A*; and min(*A*) represents the minimal feature value of gene subset *A*.

Here, the within-class distance and the between-class distance of the *k* nearest neighbor samples are calculated by the Euclidean distance, which can reflect the degree of similarity between the two data. The smaller the value is, the smaller the difference between the two data is. Since the Euclidean distance function effectively reflects the basic information of the unknown data^51^, it is introduced into this paper, and expressed as

10 $${{\rm{\Delta }}}_{A}(x,y)=\sqrt{\sum _{k=1}^{|A|}{|f(x,{a}_{k})-f(y,{a}_{k})|}^{2}},$$

where |*A*| denotes the cardinality of the genes in *A*, and *f*(*x*, *a*_*k*_) represents the value of sample *x* on gene *a*_*k*_.

**Remark 1**. To evaluate the weight values of genes for samples more effectively, all selected samples in the same class and the different class cover the entire sample dataset as evenly as possible. Since the samples used in the each iteration are all randomly selected, the sample points selected randomly may not be exactly the same as the ReliefF algorithm runs each time, even if the training samples are the same. It follows that the weight values of genes will take on fluctuation. To solve this issue, the average distance among *k* nearest or *k* non-nearest neighbor samples estimates a quantitative representation of the difference among samples, and many more samples are selected such that it is closer to the actual situation of the samples. It can be observed from Definitions 1 and 2 that the weight fluctuations are efficient, and then the calculation will be more accurate.

Note that when the weight of the important gene becomes larger, it is easily separated from the others and helpful to be selected by the ReliefF algorithm. Meanwhile, when decreasing the distance between the same samples, the distance between the different samples will be increased, so that the difference of weights is very obvious. In order to obtain the more stable results in emergencies, a new distance coefficient is proposed to further reduce the instability during calculations.

**Definition 3**. A distance coefficient is defined as

11 $$CD=\frac{\sqrt{\frac{\sum _{i=1}^{k}({x}_{i}-\overline{x})}{k}}}{\sum _{i=1}^{k}{x}_{i}},$$

where *k* is the number of genes, $\bar{x}$ is the average gene value of selected samples, and *x*_1_, *x*_2_, …, *x*_*i*_, …, *x*_*k*_ are the values of genes for the *i*-th sample.

**Remark 2**. From Definition 3, the greater the variation degree of two genes is, the larger the distance coefficient value is. The distance coefficient further reduces the instability of calculations, and makes the results more stable in emergencies.

**Definition 4**. A new formula of updating weight coefficient of genes in the ReliefF algorithm is defined as

12 $$W[A]=W[{A}_{0}]-\frac{C{D}_{same}\sum _{j=1}^{{\rm{k}}}di{\rm{s}}(A,{x}_{i},H)}{mk}+C{D}_{diff}\sum _{C\ne class({x}_{i})}\frac{{p}_{i}(C)}{1-p(class({x}_{i}))}\cdot \frac{\sum _{j=1}^{k}dis(A,{x}_{i},{M}_{j}(C))}{mk},$$

where *A* represents a gene subset of the filtered dataset; *A*_0_ is the gene set of the original dataset; *W*[*A*_0_] is the weight coefficient before updating; *CD*_*same*_ is the distance coefficient of the nearest neighbor samples in the same class; *CD*_*diff*_ is the distance coefficient of the nearest neighbor samples in the different classes; *x*_*i*_ is the *i-*th sample and *H* represents the nearest neighbor samples with *x*_*i*_ in the same class; *diff*(*A*, *x*_*i*_, *H*) is a quantitative representation of the difference between the sample *x*_*i*_ and *H* on the each gene in *A*; *m* is the number of cumulative repeats; *k* is the number of nearest neighbors; *p*(*C*) is the ratio of the target samples *C* to the total samples; *p*(*class*(*x*_*i*_)) is the ratio of the samples of classes including *x*_*i*_ to the total samples; *M*_*j*_(*C*) denotes the *j*-th neighbor sample in the different classes with the target samples *C*; and *diff*(*A*, *x*_*i*_, *M*_*j*_(*C*)) is a quantitative representation of the difference between the samples *x*_*i*_ and *M*_*j*_(*C*) on the each gene in *A*.

### Improved ACO method

In the ACO algorithm, the three important tasks of an ant search include rule generation, pruning rule, and pheromone updating, in which the pruning rule is an important process that affects the performance of the ACO algorithm^41,44,48^. The pruning rule removes the extraneous elements, which helps to avoid the overflow of the training data, and also simplifies the rules, because the simpler rules are easier to understand for users than the longer rules. Since the repeated selection of path nodes may result in an over-fitting of the classification rules for the samples, the rules are pruned after the rules are generated, so that it can improve the efficiency of ACO. In addition, the pruning rule can describe the objects with a minimum set of genes and a minimum number of classification rules to achieve the effective classification of objects. Then, a new pruning rule is described as follows.

**Definition 5**. For a given gene expression dataset, and any gene subset *A* with the weight coefficient *W*[*A*] of genes in *A*, the average value of weight coefficient of *A* is expressed as $\frac{W[A]}{|A|}$. Then, the genes in *A* are preliminarily selected according to the following pruning rule: When the weight value of gene is greater than $\frac{W[A]}{|A|}$, the gene can be reserved; otherwise the gene should be deleted.

**Definition 6**. The probability formula of the next point in the path selected by the ants is defined as

13 $${p}_{ij}^{k}(t)=\frac{{\tau }_{ij}^{\omega }(t){\eta }_{ij}^{r}(t)}{{\sum }_{s\in allowe{d}_{k}}{\tau }_{is}^{\omega }(t){\eta }_{is}^{r}(t)},$$

where *r* as the Pearson correlation coefficient is calculated by ${r}_{x,y}=\frac{\sum (x-\overline{x})(y-\overline{y})}{\sqrt{\sum {(x-\overline{x})}^{2}{(y-\overline{y})}^{2}}}$, and *ω* is the absolute value of the weight.

**Remark 3**. From Definition 6, Eq. (13) highlights the closeness of the correlation relationship between the reaction variables, and can increase the path visibility with large correlation based on the Pearson correlation coefficient. Then, the results will not deviate from the real-world gene expression dataset, and have the better rationality.

Note that the ants in the ACO algorithm are more inclined to choose a path with a larger amount of information^16^. Then, a kind of positive feedback mechanism is formed as follows: When the amount of information on the optimal path becomes larger and larger, the amount of information on the other paths is gradually decreasing with time. The convergence of ACO to an optimal solution is the dynamic realized process of the positive feedback of the pheromone. Thus, the pheromone adjusting strategy has a great influence on the convergence and the efficiency of the ACO algorithm. In order to increase the pheromone concentration of important genes, the pheromone left by the ants is prevented from being overwhelmed with time, and a new pheromone updating formula in ACO is adopted as follows.

**Definition 7**. A new pheromone updating formula is defines as

14 $${\tau }_{ij}(t+{\rm{\Delta }}t)=(1-\rho ){\tau }_{ij}(t)+{\rm{\Delta }}{\tau }_{ij}(t)+W[\{j\}],$$

where ${\tau }_{ij}(t+{\rm{\Delta }}t)$ is the value of pheromone updating of the *i*-th gene and the *j*-th gene; *ρ* is the retain ability of the path and stands for the attenuation degree of the path, and 0 < *ρ* < 1; *W*[{*j*}] represents the weight coefficient of the *j*-th gene, which can increase the pheromone concentration of important genes; and *τ*_*ij*_ denotes the pheromone on the edge (*i*, *j*). Since the amount of information on each path is equal, *τ*_*ij*_(0) = 0 at the initial moment, the ant traverses each gene point according to Eq. (13), and after the steps are executed, the pheromone is updated for all gene points according to Eq. (14).

**Remark 4**. From Definition 7, the weights are introduced here to make the calculation of pheromone concentration more accurate, and it can eliminate the interference of the difference data as much as possible. Then, the operation process of pheromone updating is more stable and the operation result is more accurate. Thus, based on Definitions 5–7, the improved ACO algorithm has the ability of the strong positive feedback, and it quickly converges to an optimal solution through the accumulation and updating the pheromone.

### The RFACO-GS algorithm

**Figure 1:**
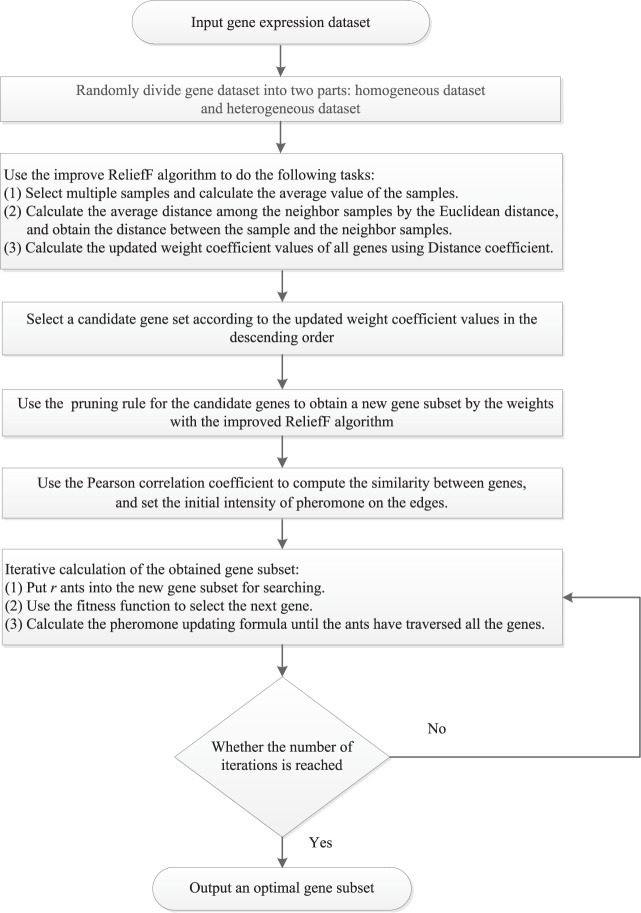
The detailed flowchart of the proposed RFACO-GS algorithm.

Since there are too many gene types that have few relevant genes in gene expression datasets, this paper proposes a hybrid filter-wrapper method for gene selection to solve these existing problems. Then, a ReliefF and ACO-based gene selection (RFACO-GS) algorithm is designed in this subsection. The detailed flowchart of the proposed RFACO-GS algorithm is shown in Fig. 1. It should be noted that, following the experimental techniques designed by Wei *et al*.^52^ and Li *et al*.^53^, the gene expression dataset will be divided into two parts including homogeneous dataset and heterogeneous dataset, where many samples are randomly selected; their average value of sample genes are calculated, denoted as $\bar{x}$; and *k* nearest neighbor samples in the same class and *k* nearest neighbor samples in the different classes are obtained, respectively. It follows from Remark 1 that the selected samples can cover each sample category as evenly as possible by using the average of the samples instead of the randomly selected samples. Thus, this state is closer to the real situation of the dataset, and can avoid the contingency of randomly selecting only one sample. This step can make the calculation more precise, and can eliminate the weight fluctuation caused by the random selection of the samples.

As can be seen from Fig. 1, for the gene expression dataset, some unrelated genes are firstly excluded, and the improved ReliefF algorithm is adopted to calculate the weights of the strong correlation genes for classification. According to the results sorted backward, the irrelevant genes can be filtered out, and then the genes with the high correlation of classification characteristics are obtained. Secondly, the genes with large-weights are ordered and selected according to the weights calculated by the improved ReliefF, and the improve ACO algorithm is used to prune rule for the candidate gene subset. Finally, after one search, the top several genes are sorted in descending order according to the weights for the next search, and by iteration, the gene subset with the highest classification accuracy are obtained ultimately as an optimal solution. To facilitate the understanding of the RFACO-GS algorithm for the gene expression datasets, the special steps of RFACO-GS algorithm are described as follows.

**Algorithm 1:**
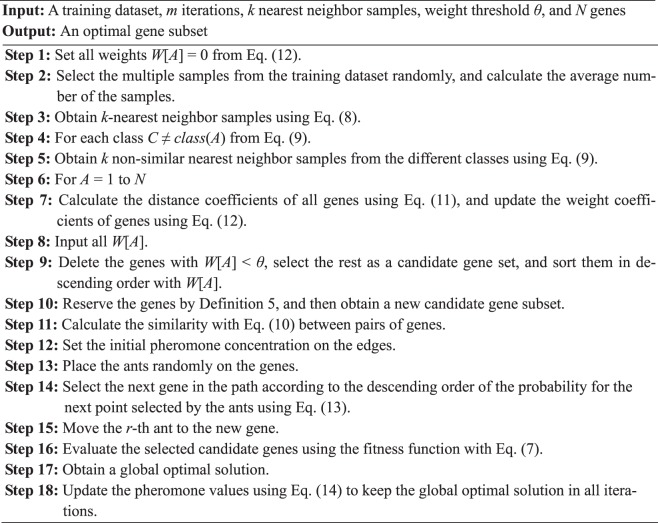
RFACO-GS.

## Experimental Results and Analysis

### Experiment preparation

In this section, we conduct the extensive experiments to verify the classification performance of the proposed RFACO-GS algorithm, and then the simulation experiments are performed on six public gene expression datasets, which can be downloaded at http://bioinformatics.rutgers.ed/Static/Supplemens/CompCancer/datasets. The descriptions of the six gene expression datasets are shown Table 1. It can be seen form Table 1 that the number of samples is between 63 and 203, and the number of genes is between 2000 and 12600. So these data are typical high-dimensional data of small samples. Following the experimental techniques of parameter setting^54,55^, the detailed parameters in the RFACO-GS algorithm are described as follows: the number of ants is *r* = 100 in Algorithm 1, the maximum number of iterations is set as 80, and since the amount *Q* of pheromone on the path from ants in iterations is related to the distance between notes *i* and *j*^56^, one sets *Q* = 100 in Eq. (6). The experimental operating system is Windows 7 with an Intel Core i55200U at 1.50 GHZ, and 4.0 GB memory. All simulation experiments are implemented in MATLAB R2014a and WEKA 3.8.

**Table 1:** Overview of the six public gene expression datasets.

Datasets	Genes	Samples (category)	Classes	Author
Colon cancer	2000	62(Tumor(40), Normal(22))	2	Sun *et al*.^3^
Leukemia	7129	72(ALL(47), AML(25))	2	Chen *et al*.^37^
Prostate	12600	136(Tumour (77), Normal (59))	2	Sun *et al*.^14^
Lung	12600	203(Adeno(139), NORM(17), Squamous(21), COID(20), SMCL(6))	5	Liu *et al*.^6^
Brain	12000	50(Tumour (20), Normal (30))	2	Li *et al*.^40^
SRBCT	2308	83(EWS(29), BL(11), NB(18), RMS(25))	4	Lu *et al*.^32^

### Comparison of classification performance of related relief algorithms

This portion of our experiments evaluates the classification performance of our proposed algorithm in terms of the classification accuracy and the number of selected genes. The classification accuracies of the RFACO-GS algorithm are compared with those of the state-of-the-art related Relief Algorithms on the four gene expression datasets selected from Table 1. These methods include: (1) the ReliefF algorithm^28^, (2) the mean deviation-based sample weighting versions of ReliefF algorithm^31^ (MD-SW ReliefF), (3) the ReliefF^28^ combined with neighborhood rough sets^3^ (ReliefF + NRS), and (4) the Relief-extreme learning machine algorithm^32^ (Relief-ELM). Moreover, the classification accuracy of the dimension reduction results is verified with the 10-fold cross-validation method. Following the designed experimental techniques^3,28,31,32^, the related parameters for the five models can be found in their references, and then the experimental results of classification accuracy of the five algorithms on the four gene expression datasets are shown in Table 2. Here, it is noted that the bold font indicates the optimal value in the following subsections.

**Table 2:** Classification accuracies of the five Relief algorithms on the four gene expression datasets.

Datasets	ReliefF	MD-SW ReliefF	ReliefF + NRS	Relief-ELM	RFACO-GS
Colon cancer	78.8%	78.4%	56.4%	64.8%	**94.0%**
Leukemia	91.5%	94.4%	56.3%	65.2%	**95.8%**
Lung	96.2%	94.1%	91.9%	54.4%	**99.5%**
Prostate	93.3%	**96.4%**	64.2%	57.9%	89.2%
Average	90.0%	90.8%	67.2%	60.6%	**94.6%**

According to Table 2, the classification accuracy of the RFACO-GS algorithm is larger than the other related Relief algorithms, and nearly 40% higher than the other algorithms. Meanwhile, it is obvious that the accuracy of the Relief-ELM algorithm is the worst because all of its accuracies are lower than 65.5%. The classification accuracies of RFACO-GS on the Colon cancer, Leukemia and Lung datasets are the highest than those of the other four algorithms, except for the Prostate dataset, on which the RFACO-GS algorithm is slightly lower than the ReliefF and MD-SW ReliefF algorithms in accuracy. The reason is that our algorithm has not efficiently remove noises from the original Prostate dataset. However, the ReliefF and MD-SW ReliefF algorithms are not stable. For example, their accuracies are 78.8% and 78.4% in the Colon cancer dataset, respectively, but the other accuracies are almost greater than 90%. The ReliefF + NRS algorithm only performs well on the Lung dataset, and the classification accuracy on the remaining three datasets is less than 70%. Furthermore, the RFACO-GS algorithm obtains the highest average classification accuracy on the four gene expression datasets. Therefore, our algorithm can significantly improve the classification performance of the selected genes on the four gene expression datasets.

The following part of this experiment describes the number of selected genes of the proposed RFACO-GS algorithm compared with four gene selection algorithms on the four gene expression datasets selected from Table 1. The state-of-the-art compared methods include: (1) the ReliefF algorithm^28^, (2) the ReliefF combined with mRMR^29^ (mRMR-ReliefF), (3) the correlation-based feature selection algorithm^57^ (CFS), and (4) the ReliefF and PSO algorithm^30^ (RefFPSO). Following the offered experimental techniques^28–30,57^, the related parameters for the four models can be found in their references, and then the number of genes selected by the five algorithms on the four gene expression datasets are illustrated in Table 3.

**Table 3:** The number of genes selected by the five algorithms on the four gene expression datasets.

Datasets	ReliefF	mRMR-ReliefF	CFS	RefFPSO	RFACO-GS
Colon cancer	17	23	22	13	**9**
Leukemia	28	31	34	24	**18**
Lung	32	45	32	33	**16**
SRBCT	21	26	26	26	**13**
Average	24.5	31.25	28.5	24	**14**

From Table 3, the RFACO-GS algorithm selects the least number of genes on the four gene expression datasets, the ReliefF, CFS and RefFPSO algorithms is similar in the number of selected genes, and the mRMR-ReliefF is the worst. For the Colon cancer dataset, the RFACO-GS exhibits the best, and the number of selected genes is less than 10. For the Lung dataset, the number of genes selected by RFACO-GS is less than half of the other methods. Furthermore, the RFACO-GS algorithm achieves the least average number of selected genes on the four gene expression datasets. Hence, it can be shown that our algorithm has the optimal performance in terms of the number of selected genes, and is an efficient dimension reduction method for the high-dimensional, large-scale gene expression datasets.

### Comparison of classification performance of related ACO algorithms

The subsection of our experiments continues testing the performance of our proposed algorithm in terms of the number of selected genes and the classification accuracy on the selected genes on the four gene expression datasets selected from Table 1. The classification performance of the RFACO-GS algorithm is compared with three state-of-the-art related ACO algorithms on four gene expression datasets selected form Table 1. The contrasted algorithms include: (1) the ACO method^36^, (2) the Ant colony optimization-selection algorithm^40^ (ACO-S), and (3) the ACO-based method^42^ (AM). Following the given experimental techniques^36,40,42^, the related parameters for the three models can be found in their references, and then the experimental results are shown in Tables 4 and 5.

**Table 4:** The number of genes selected by the four algorithms on the four gene expression datasets.

Datasets	ACO	ACO-S	AM	RFACO-GS
Colon cancer	111	69	130	**9**
Leukemia	105	84	100	**18**
Lung	163	132	144	**16**
Brain	129	87	111	**10**
Average	127	93	121.25	**13.25**

**Table 5:** The classification accuracies of the four algorithms on the four gene expression datasets.

Datasets	ACO	ACO-S	AM	RFACO-GS
Colon cancer	76.5%	81.4%	78.9%	**94.0%**
Leukemia	86.3%	91.7%	89.2%	**95.8%**
Lung	83.6%	89.2%	87.1%	**99.5%**
Brain	62.4%	70.5%	66.0%	**88.0%**
Average	77.2%	83.2%	80.3%	**94.3%**

According to Tables 4 and 5, the difference in the four methods can be clearly identified. The RFACO-GS algorithm with the least number of selected genes has the highest classification accuracy, the ACO-S algorithm is the second, and the original ACO algorithm is the worst. For the ACO-S and AM algorithm, the average number of selected genes is 93 and 121.25 on the four datasets, respectively, which is far less than the original ACO algorithm. In terms of classification accuracy, the accuracy of ACO-S and AM algorithm is more than 80%, which is higher than the original ACO method. The RFACO-GS algorithm can yield the optimal classification performance. The average number of genes selected by our method on the four datasets is 13.25, and the average classification accuracy of the RFACO-GS method is 94.3%. Thus, it can be concluded that our algorithm can not only effectively remove noises from the four gene expression datasets, but also improve the accuracy of selected genes.

### Comparison of classification performance of intelligent optimization algorithms

To further verify the classification performance of our proposed method, six state-of-the-art intelligent optimization algorithms for gene selection are evaluated in terms of the number and the classification accuracy on the selected genes on the four gene expression datasets selected from Table 1. Firstly, the RFACO-GS algorithm is compared with four selected methods, which include: (1) the original data processing method (ODP), (2) the genetic algorithm^34^ (GA), (3) the particle swarm optimization algorithm^35^ (PSO), and (4) the simulating annealing algorithm^58^ (SA). Following the designed experimental techniques^34,35,58^, the related parameters of the GA, PSO and SA models can be found in their references, and then the number of selected genes and the classification accuracy are shown in Tables 6 and 7, respectively.

**Table 6:** The number of genes selected by the five algorithms on the four gene expression datasets.

Datasets	ODP	GA	PSO	SA	RFACO-GS
Leukemia	7129	123	97	105	**18**
Colon cancer	2000	129	93	101	**9**
Lung	2880	182	148	169	**16**
Brain	12000	112	129	121	**10**
Average	6002.25	136.5	116.8	124	**13.25**

**Table 7:** The classification accuracies of the five algorithms on the four gene expression datasets.

Datasets	ODP	GA	PSO	SA	RFACO-GS
Leukemia	94.4%	87.1%	86.6%	85.7%	**95.8%**
Colon cancer	81.1%	77.6%	75.7%	78.2%	**94.0%**
Lung	84.6%	85.2%	85.0%	86.4%	**99.5%**
Brain	86.0%	64.8%	64.8%	62.4%	**88.0%**
Average	86.5%	78.7%	78.0%	78.2%	**94.3%**

According to the classification results in Tables 6 and 7, the difference among the five methods can be clearly identified. The RFACO-GS algorithm achieves the least number of selected genes and has the highest classification accuracy. The genes selected by the ODP algorithm are the original ones, and the average classification accuracy is 86.5%. The classification accuracies of the GA, PSO and SA algorithms are less than those of the ODP and RFACO-GS methods, and the number of genes selected by the GA, PSO and SA algorithms is considerably larger than that of the RFACO-GS algorithm. Thus, the classification performance of the GA, PSO and SA algorithms is not desirable. The reason is that some noises of the datasets are not fully filtered when the GA, PSO and SA methods process the gene datasets, and then this situation may reduce the classification ability of selected gene subset and decrease their accuracies. What’s more, the RFACO-GS algorithm has the highest average classification accuracy for the selected genes. Hence, it can be concluded that our algorithm achieves the optimal classification performance on the four gene expression datasets.

**Figure 2:**
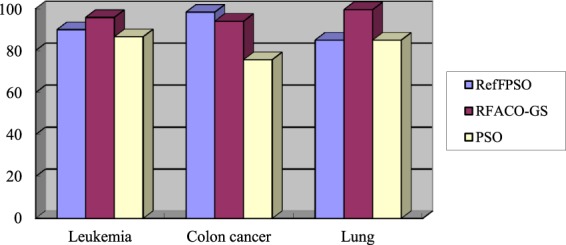
The classification accuracies (%) of the three algorithms on the three gene expression datasets.

**Figure 3:**
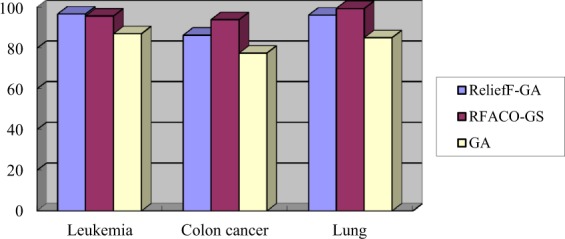
The classification accuracies (%) of the three algorithms on the three gene expression datasets.

In what follows, to illustrate the advantages of combining ReliefF with ACO, the combinations of ReliefF with PSO and GA are investigated to obtain the comparison results, respectively. Recently, Liu *et al*.^30^ proposed a gene selection algorithm combining ReliefF with PSO (RefFPSO), where the ReliefF algorithm was employed as pre-filter to eliminate the low correlated genes, and the PSO algorithm, as the search algorithm, selected the genes with high classification accuracy. Then, the experiment results in terms of the number of selected genes and the classification accuracy are shown in Table 8 and Fig. 2, respectively. Wu^59^ combined ReliefF and GA (ReliefF-GA) to study tumor classification on gene expression data, where the ReliefF algorithm selected the higher weight genes, and then the selected genes were used to guide the population initialization of GA. To clearly illustrate the comparison results, the number of selected genes and the classification accuracy are demonstrated in Table 9 and Fig. 3, respectively.

**Table 8:** The number of genes selected by the three algorithms on the three gene expression datasets.

Datasets	PSO	RefFPSO	RFACO-GS
Leukemia	97	24	18
Colon cancer	93	13	9
Lung	148	33	16
Average	113	23	14

**Table 9:** The number of genes selected by the three algorithms on the three gene expression datasets.

Datasets	GA	ReliefF-GA	RFACO-GS
Colon	129	12	9
Leukemia	123	24	18
Lung	182	32	16
Average	145	23	14

It can be seen from Table 8 and Fig. 2 that the number of selected genes and the classification accuracy of the RFACO-GS algorithm are the best for the Leukemia and Lung datasets. On the Colon cancer dataset, the accuracy of RFACO-GS is much higher than that of PSO, and slightly lower than that of RefFPSO; however, it selects only 9 genes, which greatly improves the classification efficiency. In addition, RFACO-GS has the optimal performance in terms of the average classification accuracy. In summary, these results indicate that our RFACO-GS method is indeed efficient and outperforms the PSO and RefFPSO algorithm.

According to the above experimental results of Table 9 and Fig. 3, the RFACO-GS algorithm has the least number of selected genes and the highest classification accuracy on the Colon cancer and Lung dataset, which is better than those of the GA and IReliefF-GA algorithms. On the Leukemia dataset, the classification accuracy of RFACO-GS is 0.06% lower than that of IReliefF-GA, but it can be almost ignoring, and the number of selected genes is only 9. Furthermore, the average classification accuracy of RFACO-GS is the highest. Therefore, the experimental results state that the RFACO-GS method outperforms the GA and IReliefF-GA models, and it can effectively delete the noise and achieve the better classification performance on the three gene expression datasets.

**Remark 5**. For these microarray datasets with high dimensionality and small samples, the PSO and GA algorithms are usually as randomized and population-based wrapper models, and suffer from greater computational cost and risk of overfitting for gene selection^40^. Their efficiency is much lower while the accuracy is higher than filter methods^60^. ACO has an advantage over PSO and GA of similar problems when the graph changes dynamically and the ant colony algorithm runs sequentially and can be adapt to the changes in real time^61^. In the ACO algorithm, the computational operators are simple and have no crossover and mutation, and then both memory costs and calculated time are inexpensive. When the ants in ACO proceed throughout all the search space, they can find an optimal gene combination, but PSO easily falls into the local optimal results^41^. So, ACO is particularly attractive to gene selection and has the special advantage of combination with other algorithms^37,41,44^. Since the previous ReliefF method has been initially screened, the ACO algorithm is more suitable for the initially screened genes. In addition, the ACO algorithm uses a positive feedback mechanism, is a mature convergence analysis method and can estimate the convergence speed. The algorithm of exchanging information through pheromone selection is mostly used to find the shortest path. The pheromone selection can accurately analyze the specific gravity of each gene, and the experimental results will be better. In general, it can be concluded that the combination of ReliefF and ACO algorithm can effectively produce the optimal classification performance for the high-dimensional gene expression datasets.

### Comparison of classification performance of related dimension reduction algorithms

The following section of this experiment concerns the classification performance of RFACO-GS algorithm, which is compared with the related state-of-the-art dimension reduction algorithms including: (1) the Fisher score algorithm^62^, (2) the locally linear embedding and neighborhood rough set-based gene selection algorithm^63^ (LLE-NRS), (3) the fuzzy backward feature elimination^64^ (FBFE), (4) the mutual information maximization and the adaptive genetic algorithm^32^ (MIMAGA), and (5) the distributed ranking filter approach removing the genes with information gain zero from the ranking^65^ (DRF0). Following the designed experimental techniques^32,62–65^, the related parameters for the five models can be found in their references, and then the classification accuracy and the number of selected genes are shown in Tables 10 and 11, respectively.

**Table 10:** The number of genes selected by the six algorithms on the four gene expression datasets.

Datasets	Fisher score	LLE-NRS	FBFE	MIMAGA	DRF0	RFACO-GS
Colon cancer	200	16	30	123	10	**9**
Leukemia	200	22	35	123	13	**18**
Lung	200	16	80	113	17	**16**
Prostate	200	19	50	117	113	**10**
Average	200	18.25	48.75	119	38.25	**13.25**

**Table 11:** The classification accuracies of the six algorithms on the four gene expression datasets.

Datasets	Fisher score	LLE-NRS	FBFE	MIMAGA	DRF0	RFACO-GS
Colon cancer	83.8%	84.0%	91.2%	83.8%	90.0%	**94.0%**
Leukemia	93.4%	86.8%	83.3%	**96.5%**	91.2%	95.8%
Lung	97.5%	90.7%	85.2%	94.1%	98.7%	**99.5%**
Prostate	86%	77.1%	83.2%	**97.0%**	85.7%	89.2%
Average	90.2%	83.2%	85.7%	92.9%	91.4%	**94.6%**

As shown in Tables 10 and 11, the RFACO-GS algorithm achieves the least number of selected genes and the highest average classification accuracy on the four gene expression datasets. For the Colon cancer and Lung datasets, the number of genes selected by the RFACO-GS algorithm is the least, and the classification accuracy of the selected Colon Cancer genes is the highest. But, the classification performance of the DRF0 is close to that of the RFACO-GS on the two datasets. For the Leukemia dataset, the MIMAGA algorithm has a higher classification accuracy, which is 0.7% higher than that of our algorithm, but the number of genes selected by the MIMAGA is approximately 7 times larger than that of the RFACO-GS. For the Prostate dataset, the accuracy of the MIMAGA is 7.8% larger than that of the RFACO-GS, but the number of genes selected by the MIMAGA is approximately 12 times larger than that of the RFACO-GS. Thus, our proposed algorithm exhibits the better classification performance than the other five methods on the four gene expression datasets. In summary, our proposed method can significantly reduce the dimensionality of gene expression datasets and is superior to the other related high-dimensional reduction algorithms.

## Conclusions

The identification and classification of malignant tumor genes have a wide range of applications in biology and pharmacy. In this paper, a hybrid gene selection method based on ReliefF and ACO is proposed to reduce the dimensionality of gene datasets and improve the classification accuracy. First, the ReliefF algorithm as a filter method is introduced into the distances between the sample and the samples in the same class or the different classes to effectively eliminate the weight fluctuations, and presenting a new updated weight method of genes to reduce the instability in the process of calculations. The improved ReliefF algorithm efficiently filters out genes with strong correlations with class labels. Then, a new pruning rule is designed to improve the running speed and the probability of the next point selected by the ants is defined to increase the path visibility with large correlation by introducing the Pearson correlation coefficient. A new phenotype updating method with the weight coefficient of the gene is proposed to make the operation process of pheromone updating more stable and accurate. Thus, the improved process of the ACO algorithm, as a wrapper method, can quickly converge to an optimal solution through the accumulation and the updating of pheromone. Finally, a hybrid filter-wrapper-based gene selection algorithm is developed. The experimental result shows that the proposed method is highly representative, and has less cardinality and higher classification accuracy.

In summary, the main contributions to the RFACO-GS method can be described as follows.

1. The average distance among *k* nearest or *k* non-nearest neighbor samples are introduced to more effectively evaluate the values of gene weight for samples as much as possible, so that the samples are closer to the actual situation. The distances between the sample and the samples in the same class or the different classes are defined to avoid the weight fluctuations.
2. A new distance coefficient is developed and integrated into the formula of updating weight coefficient of genes to further reduce the instability during calculations, and it is helpful to obtain the more stable results in emergencies. When reducing the distance between the same samples and increasing the distance between the different samples, the weight division is more obvious.
3. A new pruning rule is designed to reduce dimensionality and obtain a new candidate gene subset. The probability formula for the next point in the path selected by the ants is presented, which can highlight the closeness of the correlation relationship between the reaction variables, and increase the path visibility with large correlation on the basis of the Pearson correlation coefficient.
4. A new phenotype updating formula of the ACO algorithm is adopted to increase the pheromone concentration of important genes and prevent the pheromone left by the ants overwhelmed with time, and then the weights are introduced to eliminate the interference of the difference data as much as possible and make the operation process of pheromone updating more stable and accurate.

The main limitation of our proposed method is its sufficient biological explanations of the selected genes for cancer classification, and our algorithm cannot optimally balance on the size of the selected gene subset and classification accuracy in all high-dimensional gene expression datasets. Hence, the further research on the above problems will be helpful to the development of gene expression data classification. In future work, to make our algorithms more suitable for bioinformatics for biomarker discovery and to further improve the classification performance and the computational efficiency of cancer classification, new search strategies and efficient measures for biological meanings of the selected cancer characteristic genes should be explored well.

## Data Availability

The six public gene expression datasets can be downloaded at http://bioinformatics.rutgers.ed/Static/Supplemens/CompCancer/datasets. Thee datasets used to support the findings of this study are also available from the corresponding author upon request.
